# Structural insights into the enhanced carbapenemase efficiency of OXA‐655 compared to OXA‐10

**DOI:** 10.1002/2211-5463.12935

**Published:** 2020-08-08

**Authors:** Hanna‐Kirsti S. Leiros, Ane Molden Thomassen, Ørjan Samuelsen, Carl‐Fredrik Flach, Stathis D. Kotsakis, D.G. Joakim Larsson

**Affiliations:** ^1^ The Norwegian Structural Biology Centre (NorStruct) Department of Chemistry Faculty of Science and Technology UiT The Arctic University of Norway Tromsø Norway; ^2^ Norwegian National Advisory Unit on Detection of Antimicrobial Resistance Department of Microbiology and Infection Control University Hospital of North Norway Tromsø Norway; ^3^ Department of Pharmacy UiT The Arctic University of Norway Tromsø Norway; ^4^ Department of Infectious Diseases Institute of Biomedicine Sahlgrenska Academy University of Gothenburg Gothenburg Sweden; ^5^ Centre for Antibiotic Resistance Research (CARe) University of Gothenburg Gothenburg Sweden

**Keywords:** class D β‐lactamase, X‐ray, crystal structures, carbapenemases, antibiotic resistance

## Abstract

Carbapenemases are the main cause of carbapenem resistance in Gram‐negative bacteria. How β‐lactamases with weak carbapenemase activity, such as the OXA‐10‐type class D β‐lactamases, contribute to anti‐bacterial drug resistance is unclear. OXA‐655 is a T26M and V117L OXA‐10 variant, recently identified from hospital wastewater. Despite exhibiting stronger carbapenemase activity towards ertapenem (ETP) and meropenem (MEM) in *Escherichia coli*, OXA‐655 exhibits reduced activity towards oxyimino‐substituted β‐lactams like ceftazidime. Here, we have solved crystal structures of OXA‐10 in complex with imipenem (IPM) and ETP, and OXA‐655 in complex with MEM in order to unravel the structure–function relationship and the impact of residue 117 in enzyme catalysis. The new crystal structures show that L117 is situated at a critical position with enhanced Van der Waals interactions to L155 in the omega loop. This restricts the movements of L155 and could explain the reduced ability for OXA‐655 to bind a bulky oxyimino group. The V117L replacement in OXA‐655 makes the active site S67 and the carboxylated K70 more water exposed. This could affect the supply of new deacylation water molecules required for hydrolysis and possibly the carboxylation rate of K70. But most importantly, L117 leaves more space for binding of the hydroxyethyl group in carbapenems. In summary, the crystal structures highlight the importance of residue 117 in OXA‐10 variants for carbapenemase activity. This study also illustrates the impact of a single amino acid substitution on the substrate profile of OXA‐10 and the evolutionary potential of new OXA‐10 variants.

## Graphical abstract

Carbapenemases are the main cause of carbapenem resistance in Gram‐negative bacteria. OXA‐655 is a new carbapenemase variant identified from hospital wastewater. This study provides crystal structures of OXA variants bound to antibiotics to unravel the structure–function relationship and how residues impact on enzyme catalysis. The new structures illustrate how one amino acid substitution changes binding and hydrolysis of different antibiotics.

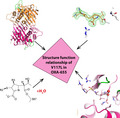

**Abbreviations**
ETP: ertapenem
IPM: imipenem
MEM: meropenem

The molecular class D β‐lactamases is a large and diverse group often referred to as oxacillinases (OXAs) due to their preference for the hydrolysis of oxacillin and related penicillins [1, 2]. Currently, more than 990 members of class D β‐lactamases have been identified (http://bldb.eu, accessed 14.01.2020). The diversity of this group of enzymes is also reflected in the heterogeneous substrate profiles of the different subgroups. These range from narrow‐spectrum to extended‐spectrum β‐lactamases, including activity towards carbapenems [1, 2]. OXA‐β‐lactamases are widespread among diverse Gram‐negative bacteria and may be either encoded in the genome of different species, or associated with mobile genetic elements such as plasmids [1, 2]. Carbapenem‐hydrolysing class D β‐lactamases (CHDLs) have predominantly been linked to particular distinct lineages (i.e. OXA‐23‐like, OXA‐24/‐40‐like, OXA‐51‐like, OXA‐58‐like, OXA‐143‐like and OXA‐48‐like) [1]. However, it has been shown that other lineages such as the OXA‐2 and OXA‐10 have weak carbapenemase activity in the same range as other CHDLs [3]. Recently, we identified OXA‐655, a novel OXA‐10 variant with enhanced carbapenemase activity compared to OXA‐10 [4]. OXA‐655 was identified in a meropenem (MEM) resistant *Escherichia coli* isolate from hospital sewage effluent at Sahlgrenska University Hospital in Gothenburg, Sweden [4]. *Bla*_OXA‐655_ was found to be located on a mobilizable IncQ1 broad‐host range plasmid. Compared with OXA‐10, OXA‐655 harbours two mutations, T26M and V117L, where V117L was suggested to be responsible for the increased carbapenemase activity. Residue 117 is found in the _115_SAV_117_ catalytic motif of OXA‐10 type enzymes [5]. The T26M mutation identified as a single mutation compared to OXA‐10 in another new variant (OXA‐656) did not result in any detectable phenotypic effects compared to OXA‐10 [4].

Biochemical characterization of OXA‐655 showed that the enhanced carbapenem hydrolysis was evident both at the cellular level and for the purified enzyme. Moreover, increased activity towards temocillin was observed. Interestingly, the V117L mutation negatively impacted the activity towards β‐lactams carrying an oxyimino R1 side chain (e.g. ceftazidime and cefotaxime (CTX)) and the catalytic efficiency against OXA and benzylpenicillin was reduced implying a functional trade‐off. In particular, the MIC for CTX was reduced 16‐fold compared to OXA‐10 in *E. coli*, which was confirmed by steady‐state enzyme kinetics where hydrolysis of CTX exhibited a 100‐fold lower *k*_cat_/*K*_m_ ratio for OXA‐655 than for OXA‐10 [4].

Molecular dynamics indicated that the V117L mutation resulted in various structural alterations that could explain the modified activity spectrum of OXA‐655 compared to OXA‐10 [4]. To further decipher the carbapenemase activity of OXA‐10 variants and the role of residue 117, we determined the crystal structure of OXA‐10 in complex with imipenem (IPM) and ertapenem (ETP) and OXA‐655 in complex with MEM (Fig. 1). To understand the functional trade‐off, we also investigated the structure–activity relationship for CTX hydrolysis in the OXA‐10 variants.

**Fig. 1:**
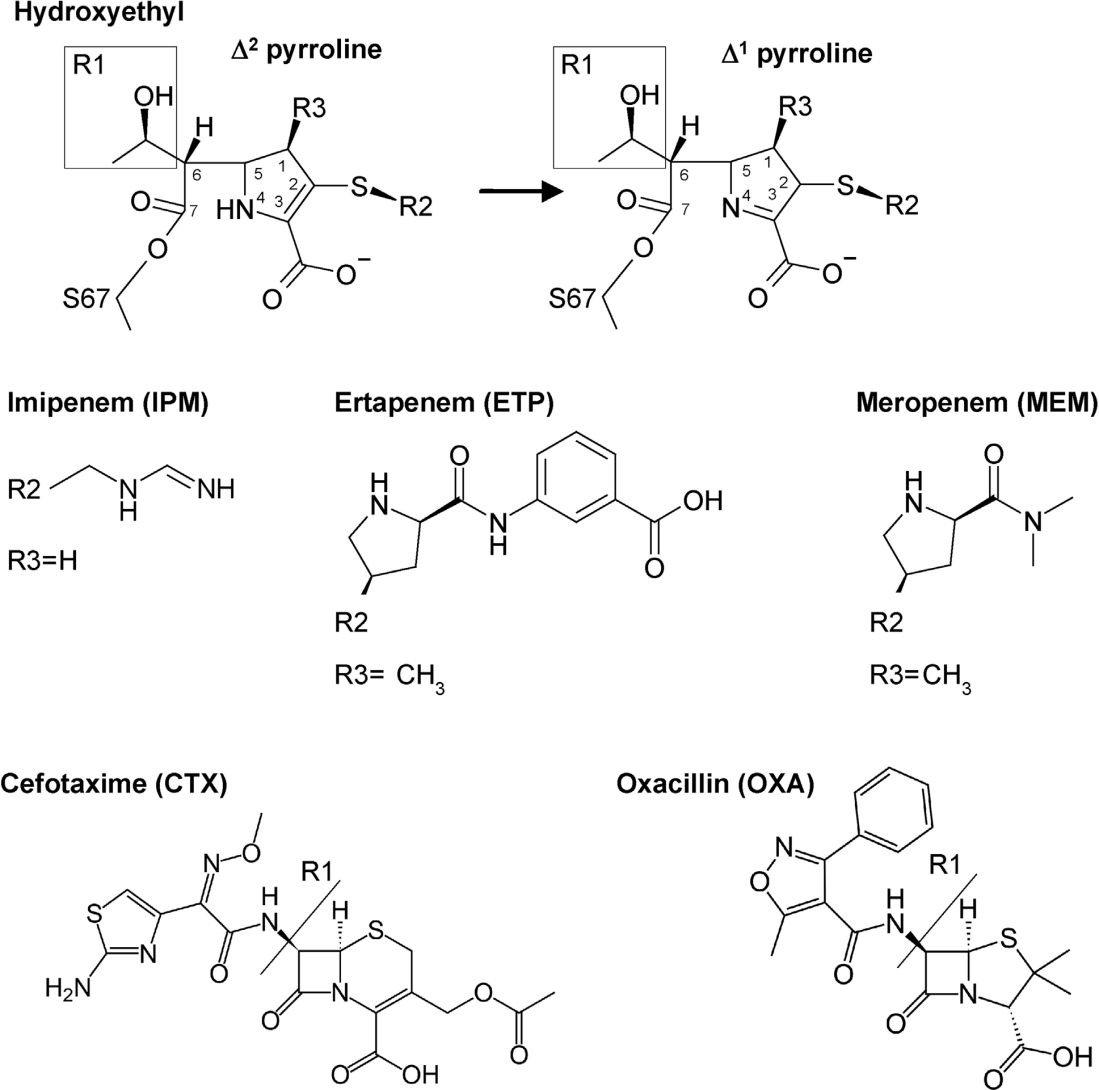
Chemical structures of a general carbapenem with the pyrroline ring in tautomer Δ^2^ (top left) and tautomer Δ^1 ^(top right), the carbapenems IPM, ETP and MEM (middle); and the cephalosporin CTX and the penicillin OXA (bottom).

## Results and Discussion

### Complex structures of OXA‐10 and OXA‐655 with carbapenems

The OXA‐10 complex structures with IPM (OXA‐10_IPM) and ETP (OXA‐10_ETP) were resolved to 1.89 and 1.85 Å resolution, respectively (Table 1, 2). Both structures were in space group P2_1_2_1_2_1_ with two molecules in the asymmetric unit denoted chain A and B. The OXA‐10_ETP structure was more ordered with lower Wilson B‐factor and lower overall B‐factors, and low *R*‐factors and *R*‐free (17.57/22.53%) compared to the OXA‐10_IPM structure (*R*‐factor/*R*‐free of 21.88%/25.11%). IPM and ETP were found in both chain A and B in the respective structures.

**Fig. 2:**
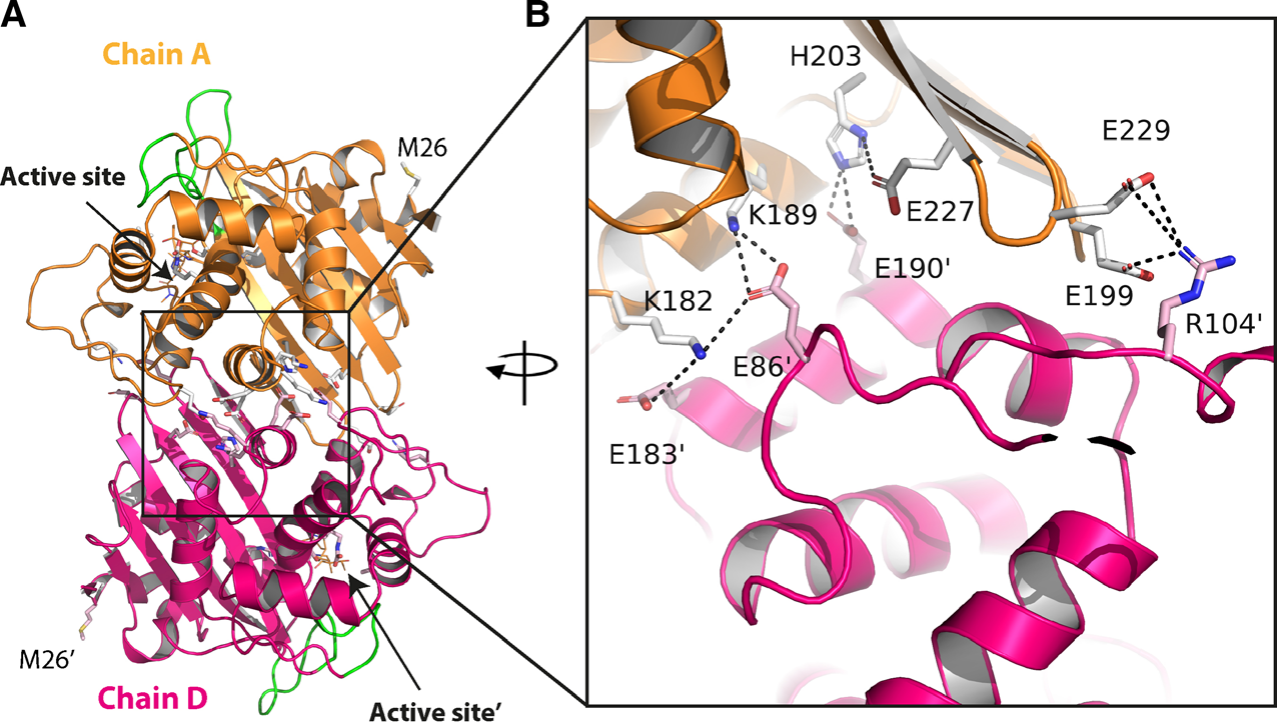
(A) Ribbon figure of the OXA‐655 dimer with chain A (orange) and chain D (pink). The omega loop (G148–G157) and the β5–β6 loop (F208–P217) are shown green, and the two active sites are highlighted. (B) Ionic networks at the dimer interface with interchain salt‐pair interactions < 4.0 Å. ‘ denotes amino acids in chain D.

The OXA‐655 complex structure with MEM (OXA‐655_MEM) was resolved to 2.10 Å in space group P2_1_ with four protein molecules (chain A/B/C/D) in the asymmetric unit and low *R*‐factor (17.70%) and *R*‐free (23.19%) values (Table 1, 2). The four molecules are arranged as two dimers, and MEM is covalently bound in an acyl–enzyme complex in all four protein chains A‐D. The overall fold of OXA‐655 was similar to OXA‐10 with the omega loop (G148‐G157) linking the α6 and β5 strand (Fig. 2A) [6] and the β5–β6 loop found adjacent to the active site.

### Overall structure and dimerization of OXA‐655

Each CHDL monomer comprises two domains, an N‐terminal helical domain and a C‐terminal mixed α/β domain with a central six‐stranded antiparallel β‐sheet [7]. OXA‐655_MEM is a homo‐dimer where the two dimers in the asymmetric unit are made up of chain A/D and B/C. When comparing the four chains in OXA‐655_MEM, the RMSD for CA atoms of chain A is 0.25/0.63/0.72 Å compared to chain B/C/D. For OXA‐655_MEM (chain A), the RMSD compared to the two new OXA‐10 structures is 0.60/0.55 Å (OXA‐10_IPM, chain A/B) and 0.53 Å (OXA‐10_ETP, both chain A and B), respectively. These values are in a similar range to RMSD scores between OXA‐10 and published structures which were identified using PDBeFOLD (https://www.ebi.ac.uk/msd‐srv/ssm/) and ranged from 0.36 Å (PDB ID: 4WZ5, chain B) to 0.62 Å (PDB ID: 1K4F, chain A).

The largest differences in OXA‐655 were observed for residues 21–22, 49–50, 93–94, 197–199 and 213–214 relative to OXA‐10_ETP and residues 264–265 relative to both OXA‐10_ETP and OXA‐10_IPM. At position 26 (Fig. 2A), the side chains of threonine in the OXA‐10 structures and methionine in OXA‐655 are all water exposed and no hydrogen bonds were identified. This explains the observation that OXA‐656, which has only the T26M mutation, shows no detectable phenotypic effects compared to OXA‐10 [4].

For all three new structures, the protein chains were arranged as dimers with a buried surface area of 1218 Å^2^ (OXA‐10_IPM), 1395 Å^2^ (OXA‐10_ETP), 1309 Å^2^ (chains A and D; OXA‐655_MEM) and 1297 Å^2^ (chains B and C; OXA‐655_MEM), found by applying the Proteins, Interfaces, Structures and Assemblies (PISA) server (http://www.ebi.ac.uk/pdbe/pisa/). Native OXA‐10 (PDB ID: 1FOF) has a cobalt ion at the dimer interface [6] bound to H203, E227 and E190’ (‘denotes residues in the other chain in the dimer). However, this metal is not found in either OXA‐655, nor in the new OXA‐10 complexes presented here and may have arisen from the inclusion of cobalt in the crystallization condition of the native OXA‐10 protein. For the OXA‐655 structure, nine hydrogen bonds and 12 ion‐pair interactions (< 4 Å) form ionic networks between residues E183’‐K182‐E86’‐K189, E190’‐H203‐E227 and E229‐R104’‐E199 (‘ denotes chain D in the dimer) (Fig. 2B) and vice versa from chain D (‘) to chain A. A similar network was observed for the chain B/C dimer interface (data not shown).

### Interactions with carbapenems in OXA‐10 and OXA‐655

Both OXA‐10 and OXA‐655 are dimers with one active site per monomer, each of which includes the motifs: _67_S‐X‐X‐K_70_, _113_S‐X‐V/L _117_ and _213_K‐T/S‐G_215_ (using OXA‐10 sequence numbering). Unique to class D β‐lactamases is carboxylation of K70, and the presence of two conserved motifs, _141_Y/P‐G‐N_143_ and _229_W‐X‐X‐G_232_, none of which are found in class A or class C β‐lactamases [7, 8]. In the three new complex structures, we found the carbapenem molecule covalently bound in all protein chains. For the two OXA‐10 structures, interactions in chain A will be described unless stated otherwise.

**Fig. 3:**
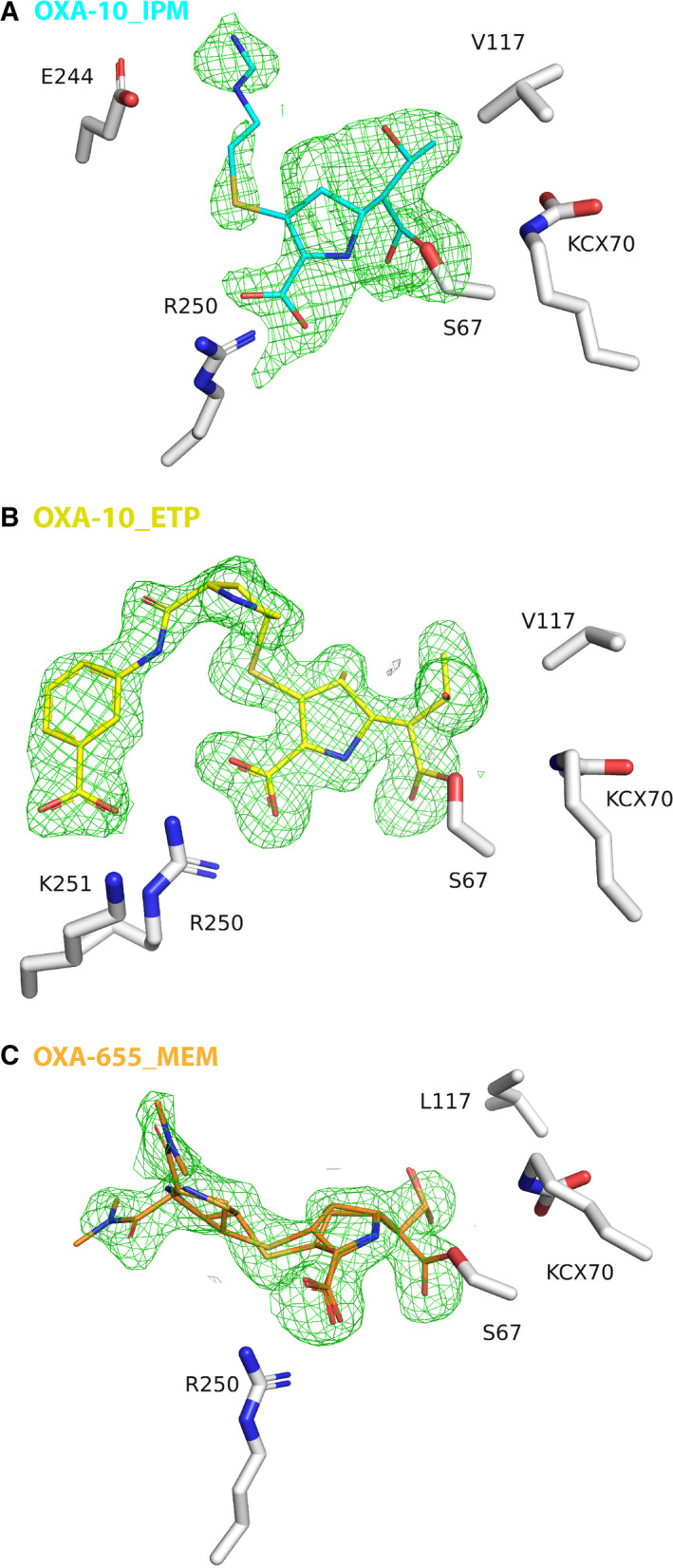
Observed electron density. Omit polder maps of (A) OXA‐10_IPM (at 2.0 σ), (B) OXA‐10_ETP (at 2.0 σ) and (C) OXA‐655_MEM (at 2.5 σ) with the complexed antibiotics covalently bound S67, and the adjacent residues V/L117, R250 and the carboxylated lysine 70 (KCX70).

The OXA‐10_IPM structure shows an acyl–enzyme complex with a clear covalent bond from the C7 carbon (Fig. 1) to the S67 OG (Fig. 3A), similar to the complex structure of OXA‐48 with IPM (PDB ID: 5QB4; [9]). The 6α‐hydroxyethyl group (R1) of IPM faces V117, L158 and W102. S115 is found in two conformations where one (occupancy 0.21) makes hydrogen bonds to S67 OG and KCX70 NZ, and the other confirmation (occupancy 0.79) forms hydrogen bonds to K205 NZ and the IPM atom N4. A strong ion‐pair interaction from the carboxylate group of IPM to R250 (< 3 Å) is observed. The carbonyl group of the ring‐opened β‐lactam ring forms hydrogen bonds with the main chain nitrogens of Y208 and S67, described as the oxyanion hole [10]. The positively charged R2 amino group of IPM is situated in a water exposed pocket defined by M99, F208, L247 and E244. E244 OE1/OE2 atoms are 3.47 Å/3.96 Å away from the positively charged R2 amino group of IPM. This R2 group is not so well defined in the omit polder map (Fig. 3A) resulting in higher *B* values.

The OXA‐10_ETP structure is also an acyl–enzyme complex (Fig. 3B) with similar interactions for the ring‐opened carbapenem core and the R1 hydroxyethyl group as in OXA‐10_IPM. The R2 tail of ETP with the pyrrolidine five‐membered ring is adjacent to M99, Q101, F208 and E244. The carboxyphenyl group of ETP stacks towards P248, and the carboxyl group is 2.8 Å away from R250 (similar to OXA‐10_IPM) and 3.6 Å away from K251. The OGA atom in the hydroxyethyl group of ETP makes hydrogen bonds with both the OG and O atoms of S115 (chain A). In chain B, the R1 hydroxyl ethyl group is rotated ~ 120 degrees, no hydrogen bond to S115 is observed, and S115 is refined in two conformations. The N atom in the pyrrolidine ring of ETP makes a water‐mediated hydrogen bond with N101, which is refined in two conformations.

**Fig. 4:**
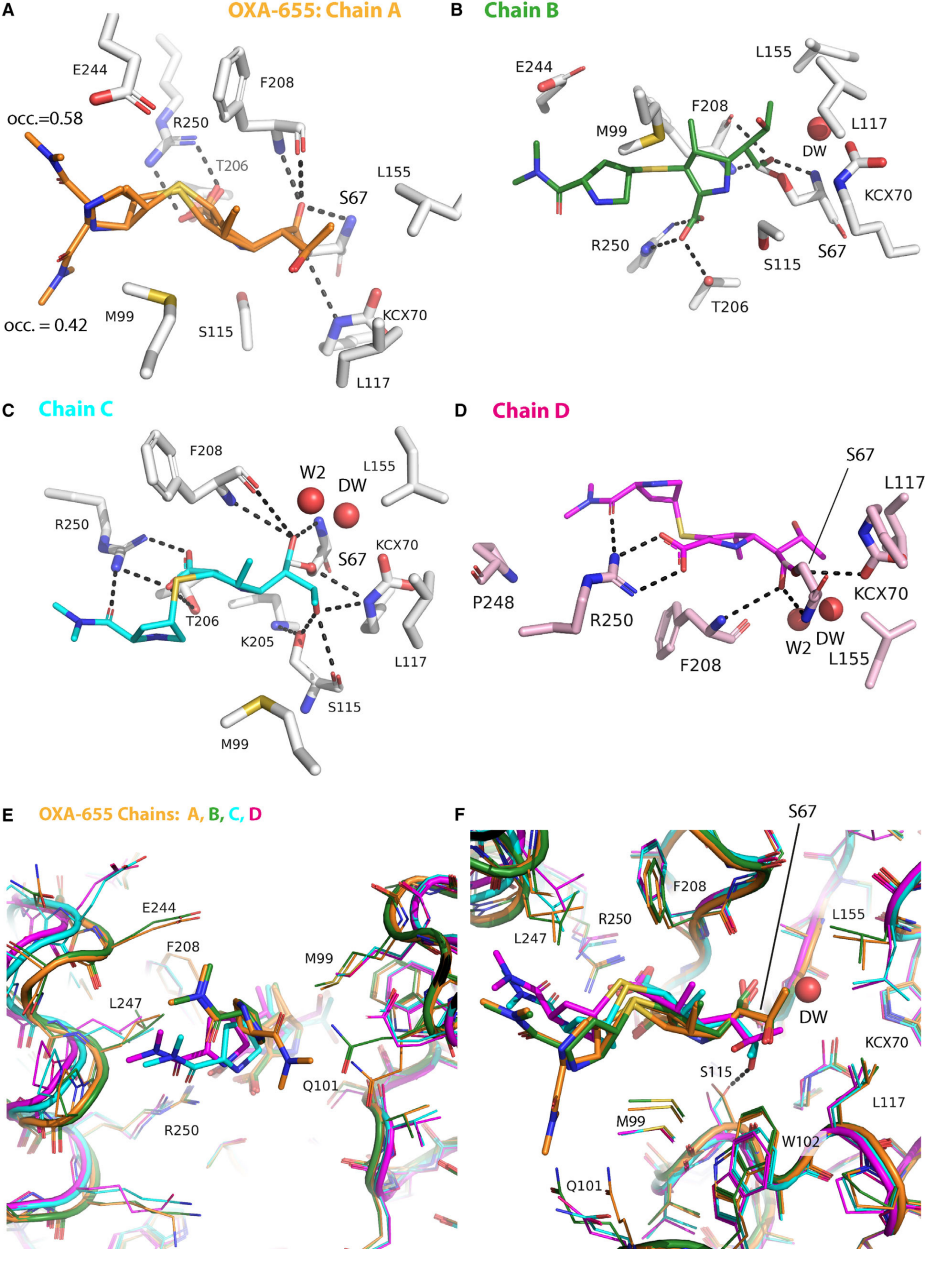
Panels A‐D show the hydrogen bonds and adjacent residues for MEM bound in chains A‐D of OXA‐655_MEM. (E, F) Superposition of the four chains A (orange), B (green), C (cyan) and D (dark pink) in OXA‐655_MEM including the MEM. The deacylation water (DW) and water 2 (W2) described in the text are shown in some panels.

In the OXA‐655_MEM structure, a covalent bond from the C7 carbon in MEM to S67 is observed (Fig. 3C) for all four chains. In chain A, MEM was refined in two conformations, the first with occupancy 0.58 which is adjacent to E244 and L247, whereas the second conformation with occupancy 0.42 is adjacent to M99 (Fig. 4A). In chain A, the Q101 side chain is disordered. The common features for both chain A conformations are as follows: (a) ionic interactions from R250 to the carboxylate group of MEM, (b) a hydrogen bond from one of the carboxylate oxygens (O12) to T206 OG1, (c) a polar interaction from S115 to N10, (d) hydrogen bonds from both S67 N and F208 N to the N6 (MEM) in the oxyanion‐hole [10] and (e) similar rotamers for the 6α‐hydroxyethyl group (R1) of MEM.

In chain B, the MEM conformation is very similar to the chain A conformation with occupancy 0.58 (Fig. 4A top; Fig. 4B), but here residue Q101 is defined in the electron density. For chain C, a third MEM conformation is present where the R1 hydroxyethyl group is rotated (Fig. 4C), so that the O8 hydroxyl can make hydrogen bonds with S115 OG (2.49 Å), S115 O (3.10 Å) and KCX70 NZ (3.15 Å). S115 OG makes a hydrogen bond (3.28 Å) with K205 NZ, and this hydrogen bond is stronger in chains A, B and D (2.61–2.77 Å). For chain C, L155 and L117 are found within Van der Waals distance of each other (Fig. 4C). The R2 MEM group has similar orientations in chains C and D (Fig. 4C,D) with a hydrogen bond from O24 to R250 NH2 (2.60 Å C) and it is facing P248, thus different from chains A and B (Fig. 4E,F).

In OXA‐655_MEM protein chain D, L155 and L117 make Van der Waals interactions with each other (Fig. 4D,F) as observed in chain C. The hydroxyl moiety of the R1 group of MEM does not participate in any hydrogen bonds and has a slightly different rotation compared to the other chains (Fig. 4E,F), while the R2 group makes a hydrogen bond from MEM O24 to R250 NH1 (Fig. 4D) as in chain C.

After superposition the chains A–D (Fig. 4E,F), the common interactions to MEM are: a covalent bond to S67 OG, an ionic interaction to R250, a hydrogen bond to T206 OG1 and hydrogen bonds in the oxyanion hole. For chain C only, O24 in the R2 group of MEM makes an additional hydrogen bond to R250, while in both C and D the R1 hydroxyl (O8) forms three hydrogens bonds. The three different rotamers and interaction types show an adaptable OXA‐655 protein, which is needed during any enzymatic cycle.

### The role of residue 117 in OXA‐655 and OXA‐10

Position 117 is the third residue in the _115_S‐A‐V_117_ catalytic motif of OXA‐10 type enzymes which is equivalent to the _130_S‐D‐N_132_ motif of class A β‐lactamases [5]. Residue 117 is valine in OXA‐10 and leucine in OXA‐655, and molecular dynamics simulations suggested that this mutation is the main contributor to the phenotypic changes observed for OXA‐655 [4].

Superposition of monomers A/B/C/D in the OXA‐655_MEM crystal structure shows that L117 has the same rotamer conformation for all four chains, but L155 makes Van der Waals contacts with either the C9 atom in the R1 hydroxyethyl group of MEM (chain A/B) or the L117 (chain C/D) (Fig. 4A,B). The distance from F208 (CD2‐atom) to M99 (SD‐atom) is 8.2/8.0/10.2/10.3 Å in chain A/B/C/D. Residue 117 is solvent exposed with a water accessible surface area of 36/31/38/44 Å^2^ in OXA‐655_MEM chain A/B/C/D, compared to 23/12 Å^2^ in OXA‐10_IMP A/B and 23/21 Å^2^ for OXA‐10_ETP A/B for V117 in the OXA‐10 structures. In addition, the mean water accessible surface area of OXA‐655 (A/B/C/D) is higher than in OXA‐10 (OXA‐10_IPM A/B, OXA‐10_ETP A/B) for both S67 (18.0 vs 15.2 Å^2^) and KCX70 (2.8 vs 0.6 Å^2^). Thus, having a leucine at position 117 increases the water exposure and water accessible surface area of both serine 67, which performs the nucleophilic attack, and KCX70, which is the general base [11, 15] during β‐lactam hydrolysis.

**Fig. 5:**
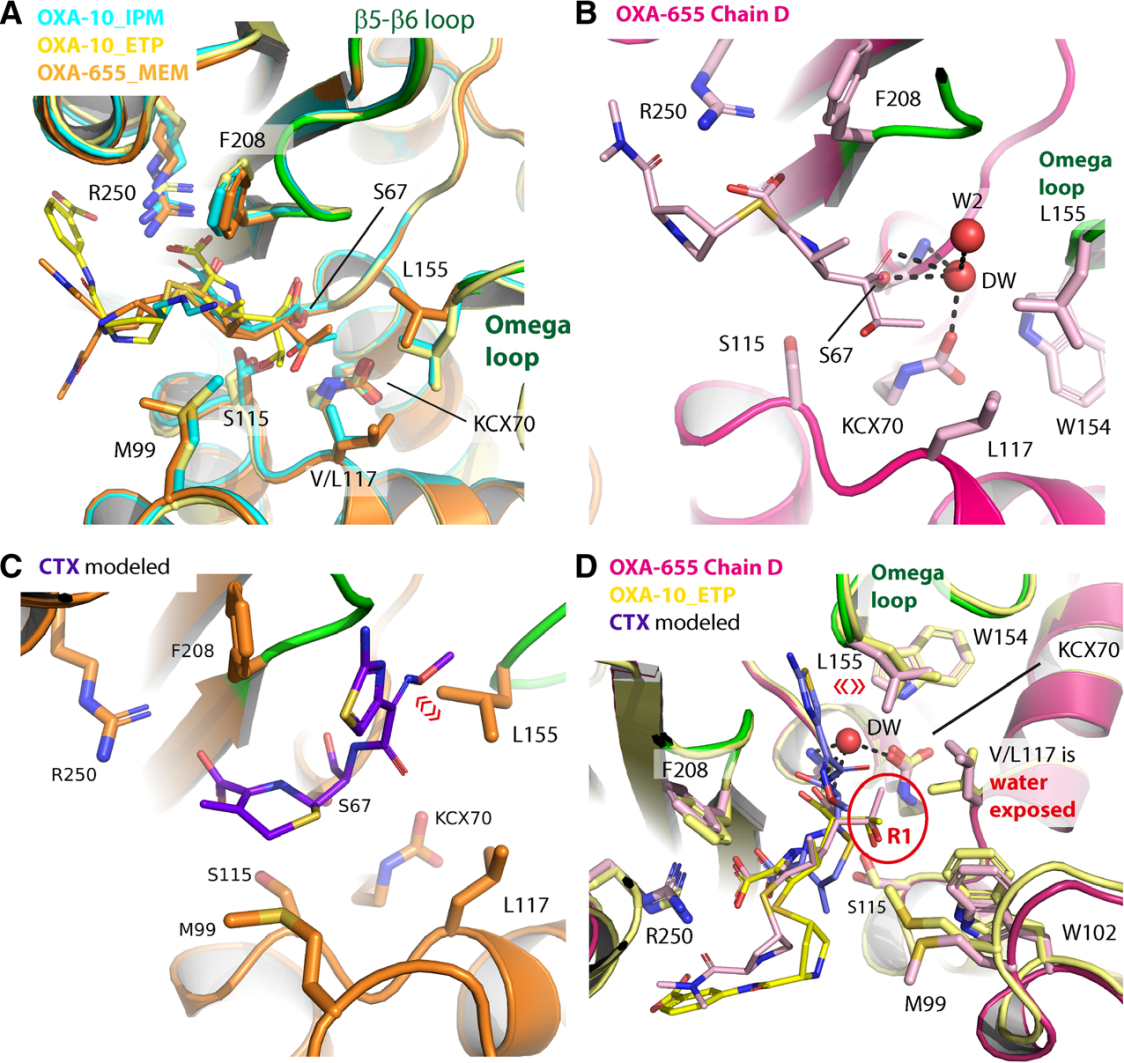
(A) Superposition of OXA‐10_IPM (cyan), OXA‐10_ETP (yellow) and OXA‐655_MEM (orange). The omega loop (residues 148–157) and β5–β6 loop (residue 208–217) for OXA‐655_MEM are depicted in green. (B) Interactions for the deacylation water (DW) in OXA‐655_MEM chain D. W2 is a second water molecule interacting with the DW. (C) CTX (violet) modelled into OXA‐655 and the collision with residue L155 (shown with red «») in the omega loop is evident. (D) Summary of the structural implications from the V177L in OXA‐655 compared to OXA‐10 here illustrated by OXA‐10_ETP. See the main text for more details.

L155 in the OXA‐10 structures is found in two different rotamers (Fig. 5A) with three chains (OXA‐10_IMP chain A and OXA‐10‐ETP chain A/B) being similar to OXA‐655_MEM chain C/D (Fig. 5D), and one chain (OXA‐10_IMP chain B) being similar to OXA‐655_MEM chain A/B. In OXA‐10, V117 has fewer and longer‐range carbon to carbon Van der Waals interactions compared with OXA‐655; especially relative to OXA‐655_MEM chain C/D (Fig. 5B). The presence of different rotamers of L155 in both OXA‐655 and OXA‐10 indicates that both enzymes are flexible and can ‘breath’ during catalysis and adopt different conformers. Position 117 is adjacent to the carboxylated primary amine group of K70 (KCX70), which acts as the general base during β‐lactam hydrolysis by deprotonating S67, and KCX70 also facilitates binding of the deacylation water [11, 15]. The structural consequence of a leucine at position 117 (OXA‐655) compared to beta‐branched valine (OXA‐10) is the increased water accessibility of KCX70 in OXA‐655, which may alter the pK_a_ of K70. However, more efficient carboxylation due to the V117L mutation would predict an increase in hydrolysis for both carbapenems and cephalosporins, and no such differences were observed [4]. For this reason, we conclude other structural effects must be responsible for the enhanced carbapenemase efficiency of OXA‐655, which simultaneously sacrifice the cephalosporinase activity.

Cephalosporin binding was further analysed by superposition of OXA‐655_MEM (chain A,B,C,D) onto OXA‐10 covalently bound to the second‐generation cephalosporin moxalactam [5] (OXA‐10_MOX; PDB ID: 1K6R). From this comparison, it is clear that L155 (chain A, B) in OXA‐655_MEM overlaps with the moxalactam R1 group of OXA‐10_MOX. L155 in OXA‐10_MOX is more similar to OXA‐655_MEM chains C and D, but rotated further towards W154 and V117 resulting in clashes and a very short distance (2.7 Å) to the L117 side chain in OXA‐655_MEM. This underscores the importance of residue 117 in the _115_SAV_117_ catalytic motif [5]. As L117 increases the water accessible surface area of S67 and KCX70, there are stronger Van der Waals interactions to L155 in the omega loop, and both the size of L117 and the L117–L155 hydrophobic interactions could hamper enzyme movements required for binding of the bulky R1 groups in cephalosporins.

### Deacylation water

The carbamylated lysine KCX70 and residue W154 are proposed to be involved in binding to the water molecule that is required for the deacylation [11, 12]. In the new OXA‐10/OXA‐655 carbapenem complexes, the deacylation water is only present in chain B/C/D of OXA‐655_MEM (Fig. 4) and OXA‐10_ETP (chain A; data not shown). The binding pattern for the deacylation water in OXA‐655_MEM (chain B/C/D) is hydrogen bonds to S67 OG, S67 N, KCX70 OX2 and O6 on MEM; and a second water (W2) in chain C/D is at hydrogen binding distance (Fig. 4C,D). The second water makes a hydrogen bond to F208 O in chain C/D. For OXA‐655_MEM chain B, the second water is missing (Fig. 4B) possibly because the conformation of the MEM R1 hydroxyethyl group almost completely overlaps with W2.

For OXA‐10_ETP (chain A), the deacylation water forms the same five hydrogen bonds or polar interactions as in OXA‐655_MEM, and the second water (W2) is present. In all our new structures W154 NE1 forms two hydrogen bonds to the KCX70 and not to the deacylation water.

The deacylation water is adjacent to residue L155 with a distance of 2.8–3.7 Å (Fig. 4F) to the CD1 or CD2 atom of L155. Since L155 is again close to residue 117, the interplay between the deacylation water, L155 and residue 117 (V/L) might then tune the substrate profile. For carbapenems to be hydrolysed, the conformation of the R1 hydroxyethyl group must also be optimal for the deacylation step to take place. In the case of OXA‐655, we speculate that the deacylation water could be more optimally positioned to take part in the deacylation step of carbapenems, since the deacylation water is closer to L155, and L155 can be more ‘fixed’ due to Van der Waals interactions with the residue 117.

This implies that the orientation of L117 found in all OXA‐655_MEM chains leaves more space for binding of the R1 6α‐hydroxyethyl group in the carbapenem, and that the deacylation water could also be more optimal for the deacylation step of carbapenems.

### Pyrroline tautomerization

From the observed electron density in all three complex structures, it was difficult to distinguish the pyrroline tautomerization after the β‐lactam ring opening. For the Δ^1^ pyrroline tautomer, there is a C3=N4 double bond, whereas for the Δ^2^ pyrroline tautomer the C2=C3 atoms are double bonded (Fig. 1). In the presented crystal structures, the Δ^2^ tautomer is used, but the Δ^1^ tautomer is also possible and indistinguishable at this resolution. The deacylation step of the carbapenem acyl–enzyme undergoes a very slow hydrolysis since many class D enzymes form stable carbapenem acyl–enzyme complexes [4, 13]. During enzyme catalysis, it has been suggested that the two tautomeric states of the acyl–enzyme intermediate have different propensities for deacylation and bond cleavage, versus inhibition and bond retention [14]. Still, the underlying enzyme mechanism for the different deacylation rates of carbapenem acyl–enzyme complexes has not yet been elucidated. For OXA‐665, this deacylation step is likely to be faster since the carbapenem drugs are cleaved at a higher rate than for OXA‐10 [4].

### Carbapenem hydrolysis by OXA‐655

A molecular dynamics simulation of OXA‐10 and a homology model of OXA‐655 indicated that the interactions between L117 and L155 are important, and that the shorter V117 residue (in OXA‐10) cannot interact with L155. Further, stronger Van der Waals interactions between L117–L155 in OXA‐655 were proposed to enforce a stronger M99–F208 interaction.

From a structural perspective, the main causes of the increased carbapenemase activity in OXA‐655 appear to be:

- Leucine at position 117 resulting in removal of a beta branched valine residue.
- Removal of the CG2 methyl group of V117 (in OXA‐10) allowing for more space upon binding of the R1 hydroxyethyl group in carbapenems.
- The Van der Waals interactions from L117–L155 forming a hydrophobic surface which possibly shields the active site entrance and favours carbapenem hydrolysis.
- The interplay between the deacylation water, L155 and L117 possibly enhancing carbapenem deacylation.
- L117 resulting in the active site residues S67 and KCX70 being more water exposed in OXA‐655.

The ETP MIC is fourfold higher towards OXA‐655 [4] compared to OXA‐10 in *E. coli* under isogenic conditions. The structural reasons for this were investigated by superimposing ETP from OXA‐10_ETP onto OXA‐655. The resulting model of OXA‐655_ETP shows that the bulky ETP R2 side chain (Fig. 1) containing the pyrrolidine five‐membered ring and the meta‐substituted benzoic acid, can form interactions with R250 and K251, and also interact with M99, Q101, F208 and E244. These residues are all far away from position 117 and the leucine residue. Thus, the increased efficiency of ETP hydrolysis by OXA‐655 is not fully clear from the structural analysis, but the interplay between the deacylation water, L117 and L155 to the surroundings (Fig. 5D) seems to be important.

### Why is the catalytic efficiency of OXA‐655 lower for oxyimino‐substituted β‐lactams like cefotaxime and oxacillin compared to OXA‐10?

Enzyme characterization showed that the catalytic efficiency (*k*_cat_/*K*_m_) of OXA‐655 towards CTX and OXA is reduced compared to OXA‐10 [4]. When modelling CTX into the active site of OXA‐655, the oxyimino‐substituted (C=N‐OR) R1 group overlaps in space with L155 in the omega loop (Fig. 5C). This means the omega loop and L155 must reorient before CTX can bind. The observed Van der Waals interactions between L117 and L155 in OXA‐655 could hamper and restrict the necessary movements of L155 in the omega loop, and possibly explain the low enzyme activity for oxacillin and CTX. For OXA‐10, V117 has weaker Van der Waals interactions to L155 since valine is smaller (fewer atoms in the side chain), and movements in the omega loop including L155 require less reorientation energy upon substrate binding.

Oxacillin has poorer binding affinity to OXA‐655 reflected in its twofold higher *K*_m_, and is hydrolysed seven times less efficiently than by OXA‐10 [4]. After modelling oxacillin into both OXA‐655 and OXA‐10, we see that a closer contact between M99 and F208 [4] at the opposite side of the active site relative to position 117, which might hamper OXA binding in OXA‐655 only. The large and aromatic R1 group of OXA (Fig. 1) might be too big to bind into the OXA‐655 active site.

## Conclusion

Three new crystal structures of OXA‐10 and its T26M and V117L variant OXA‐655 were resolved as acyl–enzyme complexes with the carbapenems IPM, ETP and MEM. These complexes reveal different binding modes for OXA‐10 and OXA‐655, and a more fixed conformation for the C3‐carboxyl group in the ring‐opened β‐lactams when binding to R250 in the enzymes. The T26M mutation in OXA‐655 is surface exposed and seems too far away from the active site to affect the enzymatic properties of the enzyme, and thus, our analyses focused on the V117L mutation. The position of V117 in the _113_S‐X‐V/L_117_ motif and its close proximity to the active site seem crucial for the interactions with the omega loop, changes in water exposure for neighbouring residues and to allow space for the R1 hydroxyethyl groups in carbapenems (Fig. 1). The interplay between the deacylation water, L155 and residue 117 seems to affect the substrate profile. L117 is involved in defining the water accessibility of the catalytically important serine 67, which performs the nucleophilic attack, and the water accessibility of the general base KCX70 during β‐lactam hydrolysis. OXA‐655 is the first reported natural mutant of OXA‐10 with enhanced carbapenemase activity, and the new structures have provided structural insights into the role that residue 117 in particular has on the enzymatic inactivation of carbapenems as well as its lower affinity for bulky oxyimino‐substituted β‐lactams.

## Materials and methods

### Gene constructs, protein expression and purification

Expression and purification of OXA‐10 and OXA‐655 were performed as previously described [4]. In brief, OXA‐655 and OXA‐10 were expressed in cultures of *E. coli* C600Z1 clones carrying the recombinant plasmids pZE21‐*bla*_OXA‐655_ and pZE21‐*bla*_OXA‐10_. Expression of *bla*_OXA‐655_ and *bla*_OXA‐10_ where induced using anhydrotetracycline and the enzymes were subsequently purified using two steps of ion exchange chromatography. Initially, a Q‐sepharose column (Bio‐Rad, CA, USA) step followed by a S‐sepharose cation exchange (Bio‐Rad) step. Purity was assessed using SDS‐/PAGE, and enzyme concentration was determined by spectrophotometry at 280 nm using a molecular absorption coefficient of 47565 m ^−1^·cm^−1^ [calculated for the mature proteins (signal peptide 1–21) using the ProtParam tool of the ExPASy server; http://web.expasy.org/protparam/].

### Crystallization of OXA‐655 and OXA‐10

Crystallization trials of both OXA‐655 (6.8 mg·mL^−1^) and OXA‐10 (11.8 mg·mL^−1^) were performed by the hanging drop method in pregreased 24‐well plates (Hampton Research, Aliso Viejo, CA, USA) with 1000 μL reservoir volume and drops with 0.5 μL protein and 1.5 μL reservoir solution at 4 °C. The reservoir solutions contained 20–23% poly ethylene glycol (PEG) 3350 (OXA‐655/OXA‐10) and 0.2 m LiSO_4_ (OXA‐655) or 0.2 m magnesium formate (OXA‐10).

Native crystals were soaked for about 2 min in cryo solution (22% PEG 3350, 0.2 m magnesium formate/LiSO_4_ and 15% ethylene glycol) saturated with IPM (OXA‐10_IPM), ETP (OXA‐10_ETP) or MEM (OXA‐655_MEM). The solutions were centrifuged prior to use to avoid solid material affecting the protein crystals. All crystals were then flash‐frozen in liquid nitrogen.

### Data collection, structure determination, refinement and analysis

All X‐ray data were collected at BL14.1 Helmholtz‐Zentrum Berlin at the BESSY II electron storage ring. Images were indexed and integrated using xds [16], merged and scaled using AIMLESS [17]. About 2000 reflections were used for cross‐validation for all complexes, and the X‐ray data collection and processing statistics are summarized in Table 1. The phase problem was resolved by molecule replacement using the program phaser [18] and one monomer of OXA‐10 with avibactam (PDB ID: 4S2O) as search model. The refinements were done in phenix.refine [19] with manual model building in wincoot [20].

**Table 1:** Statistics for the X‐ray data collection for the new OXA‐10_IPM, OXA‐10_ETP and OXA‐655_MEM structures.

	OXA‐10_IPM	OXA‐10_ETP	OXA‐655_MEM
Diffraction source	BESSY BL14.1	BESSY BL14.1	BESSY BL14.1
Wavelength (Å)	0.9184	0.9184	0.9184
Temperature (K)	100	100	100
Detector	Pilatus 6M	Pilatus 6M	Pilatus 6M
Crystal‐detector distance (mm)	371.15	207.79	282.96
Rotation range per image (°)	0.1	0.1	0.1
Total rotation range (°)	130	200	200
Space group	P2_1_2_1_2_1_	P2_1_2_1_2_1_	P2_1_
*a*, *b*, *c* (Å)	94.93	48.39	68.14
	124.79	94.78	82.44
	48.48	125.59	99.00
α, β, γ (°)	90 90 90	90 90 90	90 97.01 90
Resolution range (Å)	45.0–1.89 (1.96–1.89)	25.0–1.85 (1.89–1.85)	25–2.10 (2.15–2.10)
Total no. of reflections	580 336 (51 160)	370 553 (19 418)	242 812 (17 796)
No. of unique reflections	46 526 (4266)	50 164 (3035)	61 827 (4483)
Completeness (%)	99.0 (92.4)	99.9 (99.8)	94.9 (92.9)
Redundancy	4.8 (4.9)	7.4 (6.4)	5.8 (1.4)
〈*I*/σ(*I*)〉	12.3 (1.1)	8.5 (1.0)	5.8 (1.5)
*R*_meas._	0.1675 (1.731)	0.168 (1.705)	0.184 (0.824)
CC _1/2_	0.997 (0.553)	0.996 (0.420)	0.979 (0.539)
Overall B‐factor from Wilson plot (Å^2^)	28.6	20.8	15.7

Published crystal structures that are similar the new OXA‐655 structure were identified using the PDBeFOLD online sever at https://www.ebi.ac.uk/msd‐srv/ssm/. The dimer interfaces were analysed in terms of buried surface area and types of inter chain interactions, by applying the PISA server (http://www.ebi.ac.uk/pdbe/pisa/).

### Modelling of ceftazidime binding in OXA‐655

Different β‐lactams were modelled into OXA‐655. For CTX, an OXA‐239 complex (PDB ID: 5WI3) [21] was applied, and for OXA, an OXA‐1 complex (PDB ID: 4MLL) [22] was applied. The complexes were superpositioned and the ligand fitted further into OXA‐655. The drug to enzyme interactions was analysed in wincoot. Even if the sequence identity between OXA‐655 and OXA‐239 (an OXA‐23 variant) is 35% (RMSD for CA atoms of 1.39 Å), and OXA‐655 versus OXA‐1 has 24% identity (RMSD for CA atoms of 1.81 Å), the fold of the proteins is the same, which supports the drug modelling.  

**Table 2:** Refinement statistics for OXA‐10 and OXA‐655. Values for the outer shell are given in parentheses.

	OXA‐10_IPM	OXA‐10_ETP	OXA‐655_MEM
PDB entry	6SKP	6SKR	6SKQ
Resolution range (Å)	45.0–1.89 (1.96–1.89)	25.0–1.85 (1.92–1.85)	25.0–2.10 (2.18–2.10)
No. of reflections, working set	46 466 (4265)	50 073 (4900)	61 782 (6070)
No. of reflections, test set	2099 (192)	2482 (263)	2426 (233)
Final *R*_cryst_	0.2188 (0.3228)	0.1757 (0.3031)	0.1770 (0.2390)
Final *R*_free_	0.2511 (0.3458)	0.2253 (0.3309)	0.2319 (0.2875)
No. of protein chains/Tot. no of residues	2/491	2/490	4/980
R.m.s.d			
Bonds (Å)	0.013	0.010	0.003
Angles (°)	1.21	1.09	0.59
Average *B* factors (Å^2^)	39.02	36.68	21.34
Protein	39.0	36.0	20.44
Chain A/B/C/D	33.2/43.5	25.6/46.3	19.4/20.2/21.3/19.9
Solvent	46.3	40.7	28.67
Ramachandran plot			
Favoured/Allowed/outliers (%)	96.5/3.3/0.2	96.9/3.1/0.0	96.5/3.2/0.3
Clashscore	8.09	7.44	3.10

## Conflict of interest

The authors declare no conflict of interest.

## Author contributions

CFF, SDK, and DGJL identified the OXA‐655 gene and purified enzymes. AMT and HKSL prepared crystals, soaked and solved crystal structures. HKSL analysed data and wrote the paper. All authors (HKSL, AMT, ØS, CFF, SDK, DGJL) took part in the planning of the study, commented on and approved the final version of the manuscript. HKSL, DGJL and ØS acquired funding.

## Data Availability

Coordinate and structure factor files for OXA‐10_IPM (PDB ID: 6SKP), OXA‐10_ETP (PDB ID: 6SKR) and OXA‐655_MEM (PDB ID: 6SKQ) are deposited and released in the Protein Data Bank (PDB). The raw X‐ray data are available from the corresponding author upon reasonable request.
